# Knowledge, Attitude, and Associated Factors towards Safe Abortion among Private College Female Students in Gondar City, Northwest Ethiopia: A Cross-Sectional Study

**DOI:** 10.1155/2020/8819012

**Published:** 2020-11-04

**Authors:** Birye Dessalegn Mekonnen, Chalachew Adugna Wubneh

**Affiliations:** ^1^Department of Nursing, Teda Health Science College, P.O. BOX: 790, Gondar, Ethiopia; ^2^Department of Pediatric and Child Health Nursing, School of Nursing, College of Medicine and Health Science, University of Gondar, P.O. BOX: 196, Gondar, Ethiopia

## Abstract

**Background:**

Women die from complications of unsafe abortion in developing countries because most have little knowledge about how to safely access to abortion care. Studies on knowledge, attitude, and associated factors towards safe abortion are limited in general and particularly among private college students. Therefore, the aim of this study was to assess knowledge, attitude, and associated factors towards safe abortion among private college female students in Gondar City, Northwest Ethiopia.

**Methods:**

An institution-based cross-sectional study was conducted from April 30, 2019, to May 30, 2019, among private college students in the Gondar town. Data were collected from 633 female students using self-administered questionnaires by a simple random sampling technique. Bivariable and multivariable logistic regression analyses were performed to identify association of dependent and independent variables using SPSS, version 20.

**Results:**

A total of 633 respondents participated in this study with the response rate of 97.7%. The majority (433 (68.4%)) of students had good knowledge about safe abortion. Older age (AOR = 2.79, 95% CI: 1.16, 7.29), urban residence (AOR = 2.42, 95% CI: 1.26, 4.35), family education (AOR = 3.18, 95% CI: 1.32, 7.06), and ever having heard about safe abortion (AOR = 4.36, 95% CI: 1.89, 10.83) were factors associated with knowledge of students on safe abortion. Regarding attitude, 361 (57%) of students had favorable attitude towards safe abortion. Age (AOR = 6.58, 95% CI: 2.71, 11.21) and urban residence (AOR = 1.51, 95% CI: 1.09, 2.21) were factors significantly associated with attitude towards safe abortion.

**Conclusion:**

More than half of the participants have good knowledge and attitude towards safe abortion, but still a significant proportion of students have poor knowledge and unfavorable attitude. Information, education, and communication programs on youth reproductive health should be provided to address topics on safe abortion for students. Forums and panel discussions on safe abortion need to be undertaken especially, among youths and students who come from rural area.

## 1. Background

Abortion is the termination of pregnancy before fetal viability, which can occur either spontaneously due to complications during pregnancy or can be induced [1]. Abortion is considered as safe when performed by persons with the necessary training and skills and in an environment meeting minimal medical standards, whereas unsafe abortion is done either by persons lacking training and skills or in an environment that lack minimal medical standards, or both [2, 3].

Globally, unintended pregnancies and complications of induced abortions are important health problems of youths; each year, approximately 20 million abortions are performed worldwide, around 95% of them are in developing countries [4]. Out of 4.2 million pregnancies, an estimated 420,000 induced abortions occurred in Ethiopia in 2016 [5].

Evidence suggests that a number of reproductive age women died daily because of complications arising from unsafe abortion throughout the world; the majority of this occurred in developing countries; many of them are young age, maybe in their teens, and are likely to have poor knowledge of safe abortion [6].

In Ethiopia, about half of all pregnancy abortions are unsafe because of inadequate information and unfavorable attitude towards safe abortion [7]. A study conducted in Ethiopia revealed that the rate of abortion among students was found to be 65 per 1000 women; and only 16% were reported to be safe abortion [8]. Evidence also indicated that both knowledge and attitude of women towards safe abortion in Ethiopia was limited [9]. In previous studies, marital status, family income, and educational status of the parents were associated with knowledge of safe abortion among students, whereas the previous residence was associated with attitude [9, 10].

Safe abortion is an important element of reproductive health care, which involves a range of medical and related health services, including counseling, contraception, and referrals to other reproductive health-care services as appropriate [3, 11, 12]. However, access to safe abortion continues to depend on several factors such as women's awareness of the law, shortage of safe abortion services provision, and sociocultural pressures [8, 13, 14].

College students are unprotected to unintentional early sexual encounters that lead to unwanted pregnancies. They are therefore exposed to seek for induced abortion and candidates to suffer its complications [15]. The students who undergo an induced abortion expose themselves to serious health risks such as hemorrhage, genital injuries, sepsis, and death [16]. These study participants were chosen because young women in the college environment are away from home for the first time and become free to experiment sex without any parental supervision, especially in the study area students in private colleges are in nondormitories. There is limited information on knowledge, attitude, and associated factors towards safe abortion in general, and particularly among private college students. Therefore, the aim of this study was to assess knowledge, attitude, and associated factors towards safe abortion among private college female students in Gondar City, Northwest Ethiopia. Thus, this study may help to give attention to the development of guidelines and resources regarding information, education, and communication (IEC) programs on youth reproductive health for college students.

## 2. Methods

### 2.1. Study Design, Setting, Period, and Participants

An institution-based cross-sectional study was conducted from April 30, 2019, to May 30, 2019, among private college students in Gondar City. This town is located in the Amhara Regional State of Northwest Ethiopia. There were four private colleges in Gondar City during the study period. The number of students from all private colleges was 5,448, of whom 3,756 were female students. Randomly selected female students in private colleges in Gondar City were included in the study. Students who were absent during the time of data collection and incapable to fill the questionnaire were excluded from the study.

### 2.2. Sample Size Determination

The sample size was determined using the single population proportion formula with the assumption of 95% confidence interval, 5% margin of error, and 74.17% of students who had a positive attitude towards safe abortion was taken from a previous study [17], and a nonresponse rate of 10% was considered. The design effect of 2 was used to account for the stratified sampling involved. The final sample size was determined to be 648.

### 2.3. Sampling Technique

To obtain the required sample size, stratified random sampling was used to select study participants. First, students were stratified by their department. Then, the sample size was proportionally allocated to all the departments in the institutions based on the number of female students in each department. The sample was collected from all departments by computer-generated simple random sampling method from a list of female students obtained from the principal office.

### 2.4. Data Collection Procedure

The data were collected using a structured and self-administered questionnaire that was designed by reviewing from previous studies [9, 10, 14, 17, 18]. The questionnaire was prepared in English, as English language is instructional media for all colleges. The questionnaire consists of all the variables that can meet the objectives of the study, which is related to sociodemographic characteristics, knowledge, and attitude towards safe abortion. Data collectors were selected from health professionals of the University of Gondar comprehensive specialized hospital. The data were collected while students were in classrooms. The completed questionnaires were checked for completeness by the investigators.

### 2.5. Study Variables and Measurements

The study assessed the knowledge and attitude of participants towards safe abortion. Students' knowledge regarding the safe abortion score was calculated out of the 8 knowledge specific questions that came up with a high internal consistency (Cronbach's alpha = 0.712). Each correct response earned one point and zero for the incorrect one based on the respondent's response. Finally, the respondent who scored greater than or equal to 60% in the knowledge questions were considered as having “good knowledge” and respondents who scored less than 60% were considered as having “poor knowledge.”

Students' attitude towards the safe abortion score was calculated out of 11 specific questions, which came up with a high internal consistency (Cronbach's alpha = 0.706). A five-point Likert scale, coded as 1 = strongly disagree, 2 = disagree, 3 = neutral, 4 = agree, and 5 = strongly agree, which then categorized as “agree” (strongly agree and agree) and “disagree” (strongly disagree, disagree, and neutral). The respondents who scored greater than or equal to the mean score were considered as having “favorable attitude” and those who scored less than the mean score were considered as having “unfavorable attitude” [19]. The independent variables were age, sex, religion, marital status, place of previous resident (either urban or rural), year of study, and income from family.

### 2.6. Data Quality Control

A pre-test of the data collection tool was carried out in 5% (33 participants) of the study participants who were female students of the Bahir Dar city private college. Training was given to the data collectors before the data collection on objectives of the study, procedure of data collection, ethical consideration, data quality control, and confidentiality. Before the beginning, the respondents were told about the objective of the study and their importance to participate voluntarily in the study. The collected data were checked for completeness and consistency, on a daily basis. Data were kept in a private protected place, and confidentiality was insured by not recording any personal identifiers.

### 2.7. Data Processing and Analysis

Data were coded and entered using EPI-INFO, version 7.2.2.2, and then transported to SPSS, version 20, for further analysis. Frequencies, percentages, and mean were computed to describe the key variables of the study. Bivariable and multivariable analyses were used to determine the association between different factors and the outcome variables. Those variables having *p*-value < 0.2 in the bivariable analysis were included in multivariable analysis, and the variables having *p*-value < 0.05 in the multivariable analysis were considered as statically significant. Odds ratios and the respective 95% confidence intervals were used to assess the strength of association between the variables.

## 3. Ethical Considerations

Ethical clearance was obtained from the research review committee of the College of Medicine and Health Sciences, University of Gondar. The objective of the study was explained to the students; then, verbal informed consent was obtained from the study participants. Students were also informed to have the right not to participate on the study, if they were not interested to be involved on it.

## 4. Results

### 4.1. Sociodemographic Characteristics of Respondents

A total of 633 respondents participated in this study, making the response rate of 97.7%. The mean age was 21.8 years (SD ± 2.99). Four hundred ninety-five (78.2%) of students reported that they were living in an urban area before they joined the college. Nearly, two-thirds (389 (61.5%)) of students were single and never been in a relationship. Concerning family education, 338 (53.4%) of them were from both illiterate parents (Table 1).

### 4.2. Knowledge of Respondents on Safe Abortion

The overall students with knowledge regarding safe abortion were 433 (68. 4%). The majority (609 (96.2%)) of the respondents had ever heard about safe abortion; of those 483 (79.3%) got information from health institutions. Unsafe abortion as a major health problem of Ethiopia was considered by 598 (80.3%) of the participants. Nearly three-fourths (452 (71.4%)) of the respondents replied that Ethiopia has an abortion law. Nearly half (315 (49.9%)) of the respondents mentioned the place where safe abortion performed was the hospital. Among respondents, 376 (59.4%) said less than 3 months of pregnancy is the preferable time to perform the safe abortion (Table 2).

### 4.3. Attitudes of Respondents on Safe Abortion

Among the study participants, 361 (57%) of respondents had a positive attitude towards safe abortion. The majority (531 (83.9%)) of the respondents disagreed that elective abortion should be legal and accessible under any circumstance. The majority (461 (72.8%)) of respondents have disagreed with the legalization of safe and voluntary abortion. More than two-thirds (435 (68.7%)) of students agreed as abortion services should be available at health center &hospital. The finding also showed that 406 (64.1%) of the respondents agreed that males/husbands should have influences in the decision of abortion (Table 3).

### 4.4. Factors Affecting Students' Knowledge towards Safe Abortion

In the bivariate analysis, the factors that found to have an association with knowledge towards safe abortion among students were age, marital status, residence, family education, year of study, and ever heard about abortion. In the multivariate analysis, age, family education, residence, and ever heard about abortion had an association with knowledge towards safe abortion.

Students who found in the age group of 25 and above were 2.79 times more likely to have good knowledge of safe abortion than those in the age group of 18-19 (AOR = 2.79, 95% CI: 1.16, 7.29). The respondents who came from the urban area were 2.42 times more knowledgeable than those living in rural areas (AOR = 2.42, 95% CI: 1.26, 4.35). Respondents with both parent literates were 3.18 times more likely to have good knowledge of safe abortion than their counterparts (AOR = 3.18, 95% CI: 1.32, 7.06). Students who ever heard about safe abortion were 4.36 times more likely to have good knowledge than those who did not hear about safe abortion (AOR = 4.36, 95% CI: 1.89, 10.83) (Table 4).

### 4.5. Factors Affecting Students' Attitude towards Safe Abortion

Age, religion, family education, residence, and marital status had an association with the attitude towards safe abortion in the bivariable analysis. All explanatory variables that were considered in the bivariate analyses were included in the multivariable logistic regression. Accordingly, the age of respondents and place of residence were remained to be significantly associated with the attitude of students towards safe abortion.

Students who found in the age group of 25 and above were 6.58 times more likely to have a favorable attitude towards safe abortion than those in the age group of 18-19 (AOR = 6.58, 95% CI: 2.71, 11.21). The students from urban residents were 1.51 more likely to have a favorable attitude towards safe abortion than those from rural residents (AOR = 1.51, 95% CI: 1.09, 2.21) (Table 5).

## 5. Discussion

Assessment of knowledge, attitude, and associated factors towards safe abortion among students is important to minimize the burdens associated with unsafe abortion. This study revealed that the overall knowledge of students regarding safe abortion was 68.4% (95% CI: 64.8, 72.4). This finding is consistent with other studies done in Kampala, Uganda [14], which documented 72.4% of students had good knowledge of safe abortion. However, this result was higher than that of studies conducted in Mekelle University, Northern Ethiopia [9], Wolaita Sodo University, Southwest Ethiopia [8], and Puducherry, India [20], where the knowledge score of students was reported as 44.1%, 38.8%, and 36%, respectively. The reason for this discrepancy might be due to differences in study participants; only first year students were included in these studies, whereas in our study, the study participants included were the first year and above. It is a fact that as the year of study increases, the level of knowledge of students also increases [21]. Moreover, the variation might be occurred due to differences in access to health information in different settings. As per the study conducted in Mizan-Tepi University, South West Ethiopia, among female students, 90.52% had adequate knowledge regarding safe abortion [17], which is higher than this finding.

In the present study, students who found in the age group of 25 and above were 2.79 more likely to have adequate knowledge regarding safe abortion than those in the age group of 18-19. This finding is in line with a cross-sectional study conducted in Kampala, Uganda [14]. The possible explanation might be the fact that as age increases, students' exposure to information regarding safe abortion could also increase.

This study also showed that students who came from the urban area were more knowledgeable than those living in rural areas. This may be because of urban dwellers who have a chance to access information and educational media. Respondents with both parent literates were more than three times more likely to have good knowledge of safe abortion than their counterparts. This finding was supported by studies done in Mekelle University, Northern Ethiopia [9], and the University of Buenos Aires, Argentina [22]. This may be because parents having higher educational background let their children know additional and important lessons besides the academic program.

This study revealed that students who ever heard about safe abortion were more likely to have good knowledge than those who did not hear about safe abortion. This finding is in agreement with other studies conducted in Mizan-Tepi University, Ethiopia [17], and South Africa [19]. The explanation for this might be that students, who have different information regarding health problems of unsafe abortion, could have increased awareness related to major obstacles for their education and other health-related problems. Furthermore, students are becoming more accessed to different mass media and getting more information about the problem, which may have provided good knowledge regarding safe abortion. In addition, students who have more access to youth-friendly services could have increased awareness related to abortion, which can further increase their knowledge about safe abortion.

In this study, 57% (95% CI: 52.6, 60.8) of students had a positive attitude towards safe abortion. This result is supported by a study done in Mekelle University, Ethiopia, which showed nearly half of the students (52.8%) have a positive attitude towards safe abortion [9]. This finding result is lower than other cross-sectional studies conducted in Mizan-Tepi University [17] and South Africa [19], which reported that 74.17% and 70% of students had a positive attitude towards safe abortion, respectively. This result is higher than a cross-sectional study conducted in the Somali Region, Ethiopia, which revealed 40.7% of students had a favorite attitude towards safe abortion [18]. The reason for this variation may be due to cultural beliefs and socioeconomic status.

According to this study, students who found in the age group of 25 and above were 6.58 times more likely to have a favorable attitude towards safe abortion than those in the age group of in 18-19. This finding is supported by a previous study conducted among medical school students in South Africa [19]. This study also showed that students from urban residents before joining college were more likely to have a favorable attitude towards safe abortion than those from rural residents. This finding is in agreement with a cross-sectional study conducted in the Somali Region [18], Mekelle University, Ethiopia [9], and Kampala, Uganda [14].

This study has some limitations; it assessed both health science and nonhealth science students together without separating them by department, and even the study touches sensitive issues, and hence, qualitative study was not done. The other limitations of the study are it does not include government-owned college and university students, and sociocultural factors were not assessed.

## 6. Conclusion

In conclusion, this study revealed that more than two-thirds of participants had good knowledge and more than half of the participants had a favorable attitude regarding safe abortion, but, still, a significant proportion of students had poor knowledge and unfavorable attitude towards safe abortion. Moreover, the age of students, residence, family education, and ever heard about abortion were factors that associated with knowledge on safe abortion. The age of students and residents had an association with the attitude towards abortion. Therefore, information, education, and communication programs on youth reproductive health should be provided to address topics on safe abortion for all college students. Forums and panel discussions on safe abortion need to be undertaken, especially among youths and students who come from rural areas. Youth-friendly service has to be expanded to the rural part of Ethiopia.

## Figures and Tables

**Table 1:** Sociodemographic characteristics of private college students in Gondar City, Northwest Ethiopia, 2019 (*n* = 633).

Variables	Frequency	Percentage
*Age*		
18-19	88	13.9
20–24	410	64.8
≥25	135	21.3

*Religion*		
Orthodox	425	67.2
Muslim	138	21.8
Protestant	43	6.7
Other	27	4.3

*Marital status*		
Single, never in relationship	389	61.5
Single, no current relationship	81	12.8
Single, in relationship	80	12.6
Married	83	13.1

*Family education*		
Both illiterate	338	53.4
One literate, one illiterate	214	33.8
Both literate	81	12.8

*Residence*		
Rural	495	78.2
Urban	138	21.8

*Ethnicity*		
Amhara	588	92.9
Other	45	7.1

*Department*		
Nursing	212	33.5
Pharmacy	143	22.6
Accounting	138	21.8
Management	94	14.8
Other	46	7.3

*Year of study*		
1^st^ year	143	22.6
2^nd^ year	207	32.7
3^rd^ and above	283	44.7

*Monthly income sends from family*		
<600	453	71.6
601–1000	143	22.6
>1001	37	5.8

**Table 2:** Knowledge regarding safe abortion among private college students in Gondar City, Northwest Ethiopia, 2019 (*n* = 633).

Variables	Frequency	Percentage
*Have you ever heard about the method of abortion?*		
Yes	609	96.2
No	24	3.8

*From where/whom have you ever heard about safe abortion? (n* = 609)		
Health institution	483	79.3
Mass media	413	67.8
Parents	216	35.5
Other	42	6.9

*Where safe abortion service conducted?*		
Hospital	315	49.8
Health centre	143	22.6
Private clinic	111	17.5
Home	53	8.4

*Will Safe abortion service reduce the risk of women*'*s reproductive health problem?*		
Yes	213	33.6
No	284	44.9
I don't know	136	21.5

*When is the preferable time to perform safe abortion?*		
Before 3 months of pregnancy	376	59.4
At any time during pregnancy	257	40.6

*Is unsafe abortion considered a major problem*, *today?*		
Yes	508	80.3
No	125	19.7

*Ethiopia has abortion law?*		
Yes	452	71.4
No	181	28.6

*For what reason is abortion legal in Ethiopia?*		
Not allowed for any reason in Ethiopia	204	32.2
If pregnancy is due to rape or incent	326	51.5
If pregnancy endangers life of the woman or fetus	264	41.7
For woman with physical/mental disabilities	359	56.7
For woman physically psychologically unprepared	455	71.9
If she is financially unable to rise the child	492	77.7
If the pregnancy is extra-marital	516	81.5

**Table 3:** Attitude towards safe abortion among private college students in Gondar City, Northwest Ethiopia, 2019 (*n* = 633).

Variables	Disagree	Agree
	*N* (%)	*N* (%)
Safe and voluntary abortion should be legal and accessible	461 (72.8)	172 (27.2)
Elective abortion should be legal and accessible under any circumstance	531 (83.9)	102 (16.1)
A woman under 18 requesting safe abortion service should terminate pregnancy	426 (67.3)	207 (32.7)
Safe abortion is acceptable if she is financially unable to rise the child	431 (68.1)	202 (31.9)
Safe abortion is acceptable to prevent mother's life or fetal anomaly	361 (57.0)	272 (43.0)
It is acceptable for a woman to choose safe abortion because of rape or incent	308 (48.7)	325 (51.3)
Provision of safe abortion after unwanted pregnancy can prevent mothers' life	271 (42.8)	362 (57.2)
Males/husbands have influences in the decision of aborting	227 (35.9)	406 (64.1)
Safe abortion services should be available at health centre and hospital	198 (31.3)	435 (68.7)
A woman has the right to terminate her pregnancy if she wishes	290 (45.8)	343 (54.2)
Adolescent students use induced abortions to terminate pregnancies	285 (45.0)	348 (55.0)

**Table 4:** Factors associated with knowledge of female students towards safe abortion, in Gondar City, Northwest Ethiopia, 2019.

Variables	Knowledge		COR (95% CI)	AOR (95% CI)
	Good	Poor		
*Age*				
18-19	63	25	1	1
20–24	286	124	1.09 (0.86, 1.48)	1.11 (0.94, 2.36)
≥25	84	51	1.53 (1.06, 2.14)	2.79 (1.16, 7.29)

*Marital status*				
Single, never in relationship	274	115	1	1
Single, no current relationship	61	20	0.78 (0.45, 1.35)	0.74 (0.49, 1.39)
Single, in relationship	45	35	1.85 (1.13, 3.03)	1.87 (0.94, 3.22)
Married	53	30	1.35 (0.82, 2.22)	1.68 (0.82, 2.45)

*Family education*				
Both illiterate	223	115	1	1
One literate, one illiterate	149	65	0.85 (0.59, 1.22)	0.91 (0.68, 1.28)
Both literate	61	20	1.64 (1.10, 3.27)	3.18 (1.32, 7.06)

*Residence*				
Rural	342	153	1	1
Urban	91	47	1.15 (0.77, 1.72)	2.42 (1.26, 4.35)

*Year of study*				
1^st^ year	99	44	1	1
2^nd^ year	93	114	2.76 (1.57, 4.14)	0.69 (0.39, 1.27)
3^rd^ and above	241	42	0.39 (0.24, 0.64)	1.29 (0.82, 2.03)

*Ever heard about abortion*				
Yes	424	185	3.82 (1.64, 8.89)	4.36 (1.89, 10.83)
No	9	15	1	1

*Monthly income sent from family*				
<600	301	152	1	1
601–1000	103	40	0.71 (0.51, 1.16)	0.93 (0.42, 2.41)
>1001	29	8	0.61 (0.24, 1.22)	0.96 (0.88, 4.29)

**Table 5:** Factors associated with attitude of female students towards safe abortion in Gondar City, Northwest Ethiopia, 2019.

Variables	Attitude		COR (95% CI)	AOR (95% CI)
	Favourable	Unfavourable		
*Age*				
18-19	78	10	1	1
20–24	219	191	6.80 (1.43, 10.47)	4.69 (0.96, 9.84)
>25	64	71	8.65 (2.13, 12.45)	6.58 (2.71, 11.21)

*Religion*				
Orthodox	309	116	1	1
Muslim	39	99	6.76 (4.41, 10.37)	2.19 (0.84, 9.25)
Protestant	6	37	16.43 (6.76, 19.9)	3.97 (0.93, 7.31)
Other	7	20	7.61 (3.12, 18.47)	1.71 (0.49, 6.08)

*Marital status*				
Single, never in relationship	198	191	1	1
Single, no current relationship	63	18	0.29 (0.17, 1.03)	2.49 (0.74, 4.30)
Single, in relationship	44	36	0.85 (0.52, 1.38)	0.78 (0.37, 1.64)
Married	56	27	0.50 (0.31, 0.82)	1.59 (0.81, 3.14)

*Family education*				
Both illiterate	184	154	1	1
One literate, one illiterate	126	88	0.83 (0.59, 1.18)	0.94 (0.55, 1.61)
Both literate	51	30	0.70 (0.43, 1.16)	1.03 (0.59, 1.81)

*Residence*				
Rural	289	206	1	1
Urban	72	66	1.29 (0.88, 1.88)	1.51 (1.09, 2.21)

## Data Availability

The data sets are available from the corresponding author upon reasonable request.

## Abbreviations

COR: Crude odds ratio
AOR: Adjusted odds ratio
CI: Confidence interval
SPSS: Statistical Package for Social Science
WHO: World Health Organization.
